# FSH-R Human Early Male Genital Tract, Testicular Tumors and Sperm: Its Involvement in Testicular Disorders

**DOI:** 10.3390/life10120336

**Published:** 2020-12-09

**Authors:** Salvatore Panza, Francesca Giordano, Daniela De Rose, Maria Luisa Panno, Francesca De Amicis, Marta Santoro, Rocco Malivindi, Vittoria Rago, Saveria Aquila

**Affiliations:** 1 Department of Pharmacy, Health and Nutritional Sciences, University of Calabria—Arcavacata di Rende, 87036 Cosenza, Italy; salvatore.panza13@gmail.com (S.P.); francesca.giordano@unical.it (F.G.); daniela.derose89@libero.it (D.D.R.); mluisa.panno@unical.it (M.L.P.); francesca.deamicis@unical.it (F.D.A.); ms83.santoro@libero.it (M.S.); rocco.malivindi@unical.it (R.M.); saveria.aquila@unical.it (S.A.); 2 Centro Sanitario, University of Calabria—Arcavacata di Rende, 87036 Cosenza, Italy

**Keywords:** male genital tract, human sperm anatomy, FSH receptor, human testis

## Abstract

The follicle-stimulating hormone receptor (FSH-R) expression was always considered human gonad-specific. The receptor has also been newly detected in extragonadal tissues. In this finding, we evaluated FSH-R expression in the human male early genital tract, in testicular tumors, and in sperm from healthy and varicocele patients. In sperm, we also studied the mechanism of FSH-R action. Immunohystochemistry and Western blot analysis showed FSH-R presence in the first pathways of the human genital tract, in embryonal carcinoma, and in sperm, but it was absent in seminoma and in lower varicocele. In sperm, FSH/FSH-R activity is mediated by G proteins activating the PKA pathway, as we observed by using the H89. It emerged that increasing FSH treatments induced motility, survival, capacitation, and acrosome reaction in both sperm samples. The different FSH-R expression in tumor testicular tissues may be discriminate by tumor histological type. In spermatozoa, FSH-R indicates a direct action of FSH in these cells, which could be beneficial during semen preparation for in vitro fertilization procedures. For instance, FSH positive effects could be relevant in idiopathic infertility and in the clinic surgery of varicocele. In conclusion, FSH-R expression may be considered a molecular marker of testicular disorders.

## 1. Introduction

The follicle-stimulating hormone (FSH), a central hormone of mammalian reproduction, is produced by the gonadotroph cells in the anterior pituitary gland and the classical target organs are the ovary and testis [1,2].

In recent years, FSH-R has been detected in extragonadal sites, including bone, monocytes, different sites of the female reproductive tract, and the liver [3,4,5,6,7,8,9]. It has been hypothesized that the extragonadal FSH-Rs play a role in various physiological processes.

Although all the reports point to Sertoli cells as the exclusive FSH target in the testis, the sites of FSH action within the human male reproductive system are not resolved yet. Since FSH-R possesses several polymorphisms able to affect receptor sensitivity and expression [10,11], the attention on this receptor has been always paid based on these issues. From several gene knockout experiments on FSH and FSH-R, it emerged that FSH-deficient male mice had small testes [12] and that FSH-R mutations are associated with variable degrees of spermatogenic failure [13].

Recently, FSH-R expression has been shown in the endothelium of blood vessels from malignant tumors and metastases, including prostate, urothelial, and breast carcinomas, and this suggests a role in neoangiogenesis [14,15,16]. Adult testicular germ cell tumors (TGCTs) are the most frequent malignant tumors in male patients aged 25–45 years, and their incidence has increased in recent years. There are two main subclasses of TGCTs: seminomas (SEs) and non-seminoma germ cell tumors (NSGCTs). SEs have histological features of primordial germ cells, whereas NSGCTs have varying degrees of differentiation (i.e., embryonal carcinoma, EC), presenting distinctive clinical features and differing in therapy and prognosis. NSGCTs tend to be metastatic at presentation and have a worse prognosis than SEs at an equivalent stage of disease. Despite general advances in the management of TGCTs, the molecular bases underlying their prevention and progression remain almost unknown.

In a normal human testis, estrogen physiological actions are mediated by estrogen receptor ERβ and highly variable ERβ expression has been reported in the different TGCTs. ERβ loss is associated with advanced tumor stage in several cancers, and previously, we showed a higher expression of ERβ in SE with respect to EC, suggesting its protective role in this tumor type [17].

The FSH biochemical action on target tissues is initiated by interaction of the hormone with FSH-R, which is a transmembrane protein belonging to the G protein-coupled receptor family. Upon FSH binding, FSH-R transduces a signal primarily via the adenylyl cyclase-cAMP-protein kinase A (PKA) pathway coupling to the G_α_s subunit [18,19], which also exerts rapid phosphorylation of the extracellular-regulated kinase (ERK1/2) [20,21,22,23]. Besides the canonical G_α_s/cAMP/PKA, alternative FSH-dependent transduction pathways have been reported involving different interacting molecules and cross-talks [19]. Thus, FSH signaling networks might result in a broad range of responses in its target cells [24].

No finding has been published on FSH-R expression in normal and neoplastic human testis tissues. In the last decade, the study of human sperm at the molecular level revealed the presence of several receptors whose expression varied in some pathological conditions, such as varicocele [25]. Few studies have raised the intriguing possibility that germ cells may exhibit FSH-R, and the presence of FSH-R in germinal cells such as in spermatozoa is controversial [26,27]. Herein, we evaluated FSH-R expression in normal human testis, ductuli efferentes, proximal caput epididymis, and in neoplastic human testis tissues and examined the expression in human sperm from normozoospermic and varicocele patients.

## 2. Materials and Methods

### 2.1. Chemicals

Vectastain Universal *Elite* ABC Kit, containing normal horse serum, was from Vector Laboratories (Burlingame, CA, USA) and diaminobenzidine chromogen (DAB) was from Zymed Laboratories (South San Francisco, CA, USA). Bovine serum albumin (BSA) protein standard, Laemmli sample buffer, pre-stained molecular weight markers, haematoxylin, eosin Y, dimethylsulfoxide (DMSO), Earle’s balanced salt solution (EBSS), and all other chemicals were purchased from Sigma-Aldrich (Milan, Italy). The acrylamide bisacrylamide was from Labtek Eurobio (Milan, Italy), and the Triton X-100, gel band purification kit, enhanced chemiluminescence (ECL) Plus Western blotting detection system, Hybond^TM^ ECL^TM^, and Hepes sodium salt were purchased from Amersham Pharmacia Biotech (Buckinghamshire, UK). FSH was from MSD Italia SRL (Rome, Italy). The cholesterol-oxidase (CHOD)-peroxidase (POD) enzymatic colorimetric kit was from Inter-Medical (Biogemina Sas, Catania, Italy). The specific inhibitor of PKA, H89 (no. 371963), was from Calbiochem (San Diego, CA, USA). The NBP2-36489 FSH-R antibody (6E8.2F5) was from Novus Biologicals USA, the Oct-4 antibody (#2750) was from Cell Signaling Technology, USA, and FSH-R (H-190) sc-13935, pMAPK, and pAkt Abs; normal mouse serum (NMS); normal rabbit serum (NRS); and goat anti-mouse and goat anti-rabbit Fluorescein isothiocyanate (FITC) conjugate Abs were purchased from Santa Cruz Biotechnology, INC (Santa Cruz, CA, USA).

### 2.2. Testicular Tissues for Immunohistochemistry (IHC) Studies

This study was conducted on formalin-fixed and paraffin-embedded tissues obtained from 4 Caucasian male patients (aged 29 to 36 years) showing testes with a granulomatous lesion without neoplastic lesions (control) and from 40 Caucasian male patients aged 19 to 65 years (25 seminoma and 15 pure embryonal carcinoma) undergoing therapeutic orchidectomy. Ductuli efferentes and proximal caput epididymis without neoplastic lesions were obtained from six adult patients with seminoma. Furthermore, 1 sample from a male patient with a Leydig cell tumor was used as negative control tissue. Human samples were archival cases provided from the Pathologic Anatomy Unit (Annunziata Hospital, Cosenza, Italy). The morphological studies were performed by haematoxylin and eosin staining.

### 2.3. Immunohistochemistry in Human Normal and Neoplastic Testicular Tissues

The immunohistochemical experiments were carried out on paraffin-embedded sections from all samples. A section of 5 μm thick after heat-mediated antigen retrieval was obtained. Immunodetection was performed on sections of 5 μm thick after heat-mediated antigen retrieval, using the specific primary antibodies anti-FSH-R (1:100), NBP2-36489 FSH-R antibody (6E8.2F5), and FSH-R (H-190:sc-13935 Ab) at 4 °C overnight. Then, biotinylated IgG was applied (1:600) for 1 h at RT, followed by avidin-biotin complex/Horseradish peroxidase (ABC/HRP). Immunoreactivity was visualized using DAB. Sections were also counterstained with haematoxylin. The sensitivity of the Abs was verified using normal rabbit serum and normal mouse serum, respectively, instead of the primary Abs.

### 2.4. Scoring System

Immunoreactivity for normal human genital ducts was scored as negative (−), weakly positive (+), moderately positive (++), or strongly positive (+++) by three independent observers. Immunoreactivity for human neoplastic tissues was scored using the Allred score [28], which combines a proportion and an intensity score. A proportion score representing the estimated proportion of positively stained tumor cells was assigned on a scale from 0 to 5. An intensity score was assigned by the average estimated intensity of staining in positive cells on a scale from 0 to 3. The proportion score and intensity score were added to obtain a total score that ranged from 0 to 8. A minimum of 100 cells were evaluated in each slide. Six serial sections were scored for each sample. The one-way ANOVA was used to evaluate the differences in the scores between SE and EC samples. The Wilcoxon test was used after ANOVA as a post hoc test.

### 2.5. Protein Extraction from Testicular Tissues

Protein extraction from formalin fixed paraffin embedded (FFPE) sections was realized according to Kawashima [29,30]. The isolation of tumoral areas was performed using laser capture microdissection (MMI Cell Cut Plus, OLYMPUS). The selected areas were transferred to 1.5 mL polypropylene microcentrifuge tubes, deparaffinized in xylene for 10 min, and rehydrated (absolute ethanol, 95% ethanol, 70% ethanol, 50% ethanol); after each incubation, the tissues were pelleted at 16,000 g for 3 min, and the incubation/centrifugation steps were repeated twice. Then the tissue pellets were weighed and homogenized in 100 volumes of Protein Extraction Buffer (PEB) (500 mm Tris–HCl pH 8.0 and 2% SDS). Samples were incubated at 90 °C for 90 min. The extracts were centrifuged for 20 min at 16,000 g at 4 °C, and the supernatants were stored at −80 °C until the Western blotting analyses (see below) were performed.

### 2.6. Semen Samples and Spermatozoa Preparations

Human semen was collected according to the World Health Organization [31] Laboratory Manual from healthy volunteer donors of proven fertility. Varicocele sperm samples of patients who consulted us for fertility investigation were also placed in the study. Reflux of blood in the pampiniform plexus was determined by palpation employing the Valsalva maneuver. In particular, samples used in this study were from patients with diagnosed varicocele of grade III (visible without palpation) on the left testis. In each experiment three different normozoospermic or three varicocele patients for each sample were pooled. Ejaculates were found to have 16 × 10^6^/mL of sperm cells, progressive motility of >32%, normally formed features of >10%, and viability of 80% both for normal and varicocele patients. The study was approved by the local medical–ethical committees and all participants gave their informed consent (prot. 22160 of 11 October 2017).

### 2.7. Processing of Ejaculated Sperm

After liquefaction, in each sample three different ejaculates were pooled both for normozoospermic or varicocele patients. A total of 30 normozoospermic and 60 patients with varicocele were used, then purified and recovered by the swim-up method [31]. The upper fraction was examined using an optical microscope equipped with a ×100 oil objective to ensure that a pure sample of sperm was obtained. An independent observer, who observed several fields for each slide, inspected the cells. After that, the samples were washed with unsupplemented EBSS medium and 10 × 10^6^ sperm for each tube were incubated in the same medium (uncapacitating medium) for 1 h at 37 °C and 5% CO_2_, with treatments (experimental) or without (control). The treatments were as follows: 1, 10, and 30 mUI/mL FSH (1 and 10 mUI/mL FSH represent the range values of FSH in human male systemic blood, while the 30 mUI/mL FSH was used to perform a dose- response curve). Furthermore, some samples were incubated with 5 nM H89 alone or combined with 10 mUI/mL FSH. When the cells were treated with the H89, a pre-treatment of 15 min was performed and subsequently 10 mUI/mL FSH were added for 45 min. H89 was dissolved in dimethylsulfoxide (DMSO, 0.01% final concentration in culture). DMSO when used as a solvent control did not induce any positive result in all the in vitro assays (data not shown).

### 2.8. Western Blot Analysis of Sperm Proteins

Swim-up-purified sperm samples, washed twice with EBSS without any addition, were incubated with or without the indicated treatments, and then centrifuged for 15 min at 5000 rpm. The pellet was re-suspended in lysis buffer (62.5 mmol/L Tris-HCl, pH 6.8; 150 mm NaCl; 2% SDS; 1% Triton X100; 10% glycerol; 1 mm phenylmethylsulfonylfluoride; 10 μg/mL leupeptin; 10 μg/mL aprotinin; 2 μg/mL pepstatin) as previously described [32]. Lysates were quantified using a Bradford protein assay reagent, and an equal amount of proteins (70 µg) with the Laemmli buffer was boiled for 5 min, separated under denaturing conditions on an 11% polyacrylamide gel electrophoresis, transferred to nitrocellulose membranes, and probed with an appropriate dilution of the primary Abs to evaluate FSH-R protein expression. NBP2-36489 FSHR antibody (6E8.2F5) is a mouse monoclonal raised against a synthetic peptide made against the human FSH-R protein sequence (between residues 1–200), and it was used at a dilution of 1:2000; FSH-R (H-190) is a rabbit polyclonal antibody raised against amino acids 1–190 of FSH-R of human origin and used at a dilution of 1:1000. The bound of the secondary Ab was revealed with the ECL Plus Western blotting detection system according to the manufacturer’s instructions. The sensitivity of the Abs was verified using NMS or NRS serum instead of the primary Abs. The protein bands were quantified by scanning densitometry (Imaging Densitometer GS-700 Bio-Rad, Milan, Italy), evaluated in terms of arbitrary densitometric units, and presented as the mean ± SEM. As a loading control, all membranes were subsequently stripped (glycine 0.2 M, pH 2.6, for 30 min at room temperature) and re-probed with anti-β-actin Ab (1:1000).

### 2.9. Immunofluorescence Assay

Sperm cells recovered from swim-up-purified sperm samples were rinsed three times with 0.5 mm Tris-HCl buffer (pH 7.5) and fixed with absolute methanol for 7 min at −20 °C. FSH-R staining was carried out and after blocking with normal goat serum (10%) [33], the same Abs used for the Western blotting analyses (1:100) were used. The sperm cells were incubated with normal goat serum instead of the primary Abs in the negative controls. The slides were examined under a fluorescence microscope (Olympus BX41, Milan, Italy), and a minimum of 200 spermatozoa per slide were scored.

### 2.10. Imaging

All images were visualized using an Olympus BX41 microscope and the images were taken with CSV1.14 software, using a CAM XC-30 for image acquisition.

### 2.11. Evaluation of Sperm Motility and Viability

Sperm motility and viability were assessed by means of light microscopy examining an aliquot of each sperm sample, which had been incubated in the absence (NC) or in the presence of increasing FSH concentrations, or with H89 alone or combined with 10 mUI/mL FSH. Sperm motility was expressed as percentage of total motile sperm, including the rapid progressive (PR) and slow progressive (NP) sperm (normal values: PR + NP > 40% as reported by the WHO [31]. In the same experimental conditions, viability was assessed by a red-eosin exclusion test using eosin Y to evaluate the potential toxic effects of the treatments. An independent observer scored 200 cells for stain uptake (dead cells) or exclusion (live cells). Sperm viability was expressed as a percentage of total live sperm. Viability was evaluated before and after pooling the samples and there were no adverse effects among the different treatments on human sperm survival.

### 2.12. Measurement of Cholesterol in the Sperm Culture Medium

Cholesterol was measured in duplicate by a CHOD–POD enzymatic colorimetric method according to the manufacturer’s instructions in the incubation medium from human spermatozoa and as previously reported [34,35]. Sperm samples, washed twice with uncapacitating medium, were incubated in the same abovementioned experimental conditions for 30 min at 37 °C and 5% CO_2_. At the end of the sperm incubation the culture media were recovered by centrifugation, lyophilized, and subsequently dissolved in 1 mL of buffer reaction. The samples were incubated for 10 min at room temperature, then the cholesterol content was measured at 505 nm. The cholesterol standard used was 200 mg/dl. The cholesterol results are presented as mg per 10 × 10^6^ number of spermatozoa.

### 2.13. Acrosome Reaction

The evaluation of the acrosome reaction was performed using Fluorescein isothiocyanate-Peanut agglutinin (FITC-PNA) [36]. Scoring of the staining was immediately assessed by an epifluorescence microscope (Olympus BX41) according to a published scoring system [37]. A minimum of 200 live sperm were examined for each treatment, and they were classified into two main categories on the basis of the FITC-PNA staining: (1) Spermatozoa stained with Propidium Iodide (PI) were considered dead cells, (2) spermatozoa stained with FITC–PNA but without PI were classified as live acrosome-reacted cells, and (3) spermatozoa without any fluorescence were considered acrosome-intact live cells. Values are expressed as a percentage of acrosome-reacted cells.

### 2.14. Statistical Analysis

The experiments for IHC, immunofluorescence, and Western blotting analyses were performed in at least four independent experiments. The data obtained from motility, viability, the CHOD–POD enzymatic colorimetric method, and the acrosome reaction (six experiments with different samples using duplicate determinations) were presented as the mean ± SEM. The differences in mean values were calculated by the one-way analysis of variance (ANOVA). The Wilcoxon test was used after ANOVA as a post hoc test.

## 3. Results

### 3.1. Morphological Analysis of Normal Human Tissues

**Figure 1:**
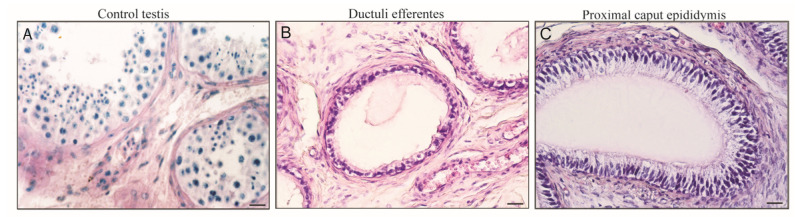
Haematoxylin and eosin staining of human control testis (**A**), ductuli efferentes (**B**), and proximal ductus epididymis sections (**C**). Scale bars = 25 μm.

The morphological analysis of control testes displayed typical seminiferous tubules showing active spermatogenesis. In the basal compartment, Sertoli cells were identified for their typical characteristics: large irregular nuclei with distinct nucleoli and extensive cytoplasmic processes extending from the basement membrane to the lumen of the tubule. Furthermore, Leydig cells were observed in the interstitial tissue (Figure 1A). Unaltered morphology of the two genital ducts was observed in our samples. Ductuli efferentes showed the typical epithelium with irregular profile, presenting groups of columnar ciliated cells alternating with groups of short non-ciliated cells. The epithelium lays on a basal lamina, which separates it from exile loose connective tissue underneath. Externally, a thin layer of smooth muscle cells is present (Figure 1B). The proximal caput epididymis revealed a regular lumen lined by a pseudostratified columnar epithelium in which principal and basal cells are evidenced. In this region a major thickness of smooth muscle cells respect ductuli efferentes is present (Figure 1C)

### 3.2. FSH-R Immunolocalization in Normal Human Tissues

**Figure 2:**
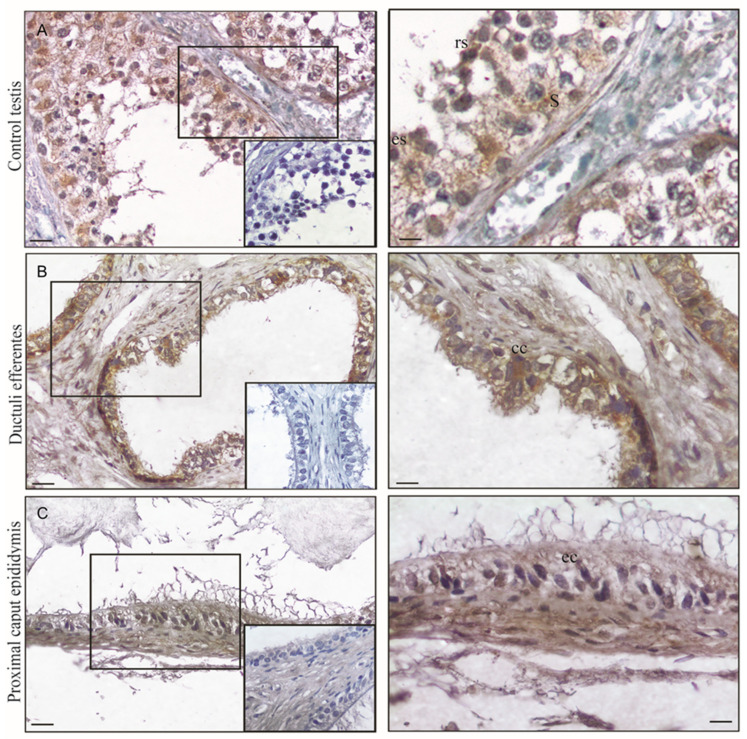
FSH-R immunolocalization on human testis (**A**), ductuli efferentes (**B**), and proximal ductus epididymis (**C**) sections. The images on the right of the figure represent the higher magnifications areas displayed in the boxes. Inserts: negative controls. Scale bars = 25 μm and 12.5 μm, respectively. S: Sertoli cell; rs: round spermatids; es: elongated spermatids; cc: ciliated cells; ec: epithelial cells.

In the testes, the immunohistochemistry for FSH-R revealed the presence of FSH-R prevalently in Sertoli cells. Particularly, it is visible on the membranes of the Sertoli cells. Interestingly, the cells of late stages of spermatogenesis (round and elongated spermatids) showed cytoplasmic immunoreactivity (Figure 2A) (Table 1). The control sections were immunonegative (Figure 2A, insert), confirming the immunostaining specificity. The same results were obtained with both primary antibodies.

When the immunostaining for FSH-R was performed in the first pathways of the human genital tract, a strong immunoreaction was observed in the epithelial cells of ductuli efferentes, particularly in columnar ciliated cells (Figure 2B), whereas in the proximal caput epididymis the epithelial layer was moderately stained (Figure 2C). Furthermore, in both regions the muscle coat showed a very weak immunostaining (Table 1). The control sections were immunonegative (Figure 2B,C inserts) confirming the immunostaining specificity.

### 3.3. Morphology of Testicular Germ Cell Tumors

**Figure 3:**
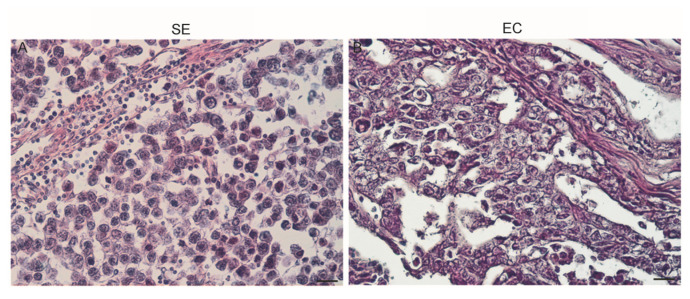
Morphology of human testicular seminoma (**A**) and embryonal carcinoma (**B**) sections. Scale bars = 25 μm.

Seminoma (SE): The classic histologic appearance of seminoma cells was observed. Large cells with prominent cell membranes contained clear cytoplasm and a hyperchromatic nucleus with a prominent nucleolus. Syncytiotrophoblasts of giant cells were evidenced. The tumor cells are arranged in small clusters separated by connective tissue septae into sheets or cords. Frequently, there is a lymphocytic infiltrate, and macrophages are often present (Figure 3A).

Embryonal carcinoma (EC): This tumor demonstrates distinctive sheets, glands, and papillary structures composed of primitive epithelial cells with crowded pleomorphic nuclei. The cells of embryonal carcinoma are arranged in several architectural patterns, and often with more than one pattern. A solid pattern with solid sheet-like arrangement and usually multiple foci of necrosis was observed. In these tumors a tubular pattern is predominant, and the cells form true glandular structures or display true papillary formations. Fibrous septations are rare, as are lymphocytic infiltrates and granulomatous reactions (Figure 3B).

### 3.4. IHC for FSH-R Localization in Human Neoplastic Testicular Tissues

**Figure 4:**
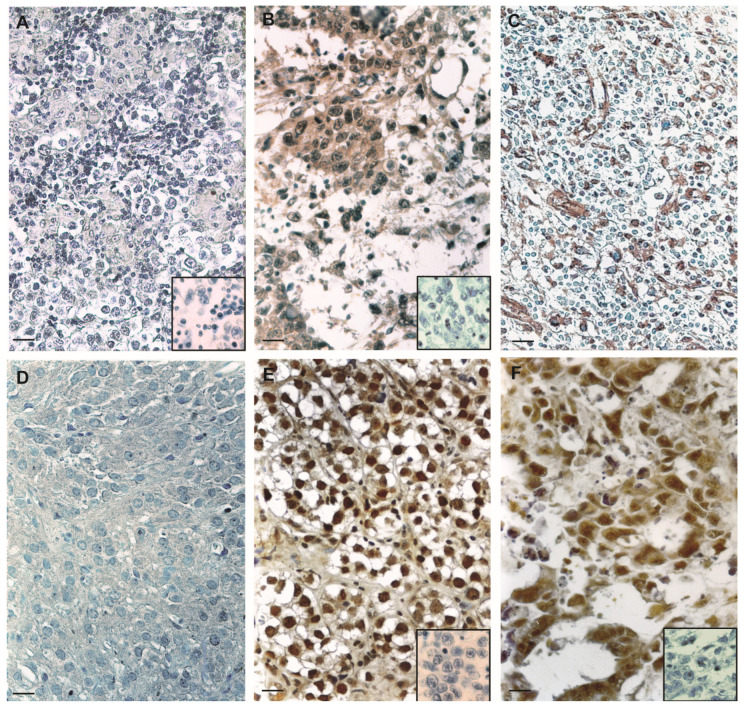
FSH-R and ER immunolocalization in human testicular sample sections. Inserts: negative controls. Scale bars = (**A**,**B**,**D**–**F**) 25 μm and (**C**) 50 μm.

Recently, it was reported that FSH-R is expressed in the vascular endothelium of a wide range of tumors, including prostate cancer, urothelial carcinoma, and renal cell carcinoma [14]. In our previous studies we evidenced that ERβ is expressed both in human testis and in SE sections as well as in the SE cell line [17,38]. Therefore, we compared the expression of ER and FSH-R in testicular germ cell cancers. Herein, we discovered that FSH-R was absent in SE and strongly expressed in EC (Figure 4A,B). Conversely, immunostaining for ERβ revealed higher intensity in SE when compared to EC (Figure 4E,F) (Table 2). Furthermore, in some tumor samples we evaluated FSH-R immunolocalization in vascular endothelium in areas adjacent to the tumor lesion (Figure 4C), whereas for the samples with Leydig cell tumors we used a negative control tissue (Figure 4D).

### 3.5. FSH-R Protein Expression in Human Normal Testicular Tissue, SE, and EC by Western Blot Analysis

**Figure 5:**
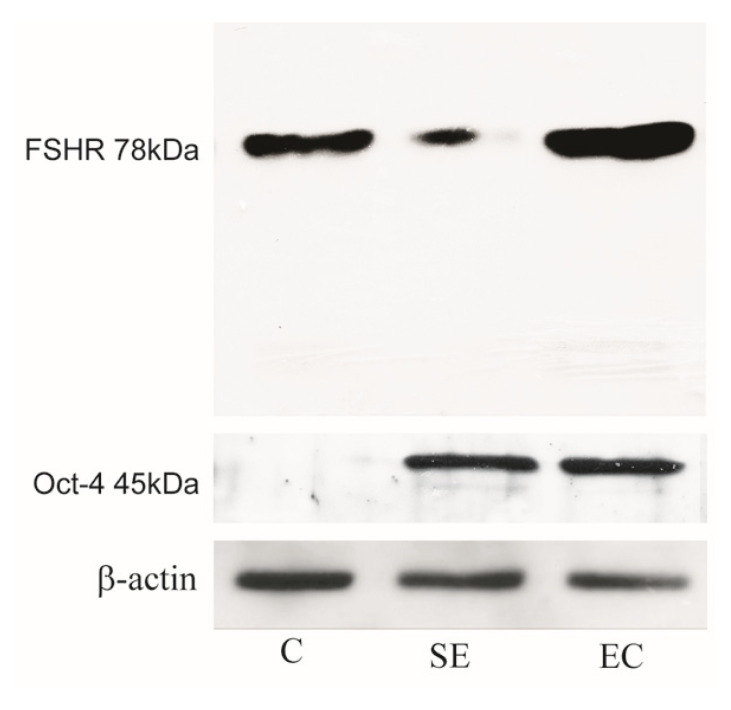
FSH-R expression is higher in EC. Representative FSH-R immunoblots of protein extracts from testicular samples. Immunoblot with the same loaded samples but with Oct-4 primary Ab used to confirm quality of the protein extracts. Actin serves as a loading control. The number given on the left represents the FSH-R molecular weight. Control testis (lane C), seminoma (lane SE), embryonal carcinoma (lane EC).

To better define the IHC data in normal and neoplastic testicular tissues, we performed Western-Blot (WB) analysis. As shown in Figure 5, FSH-R is expressed more in EC than in SE, addressing a detrimental role of this receptor in testicular cancers. Furthermore, to confirm the quality of the protein extracts, the expression of Oct-4, a specific marker of testicular germ cell cancer, was examined (Figure 5).

### 3.6. FSH-R is Present in Human Ejaculated Spermatozoa

**Figure 6:**
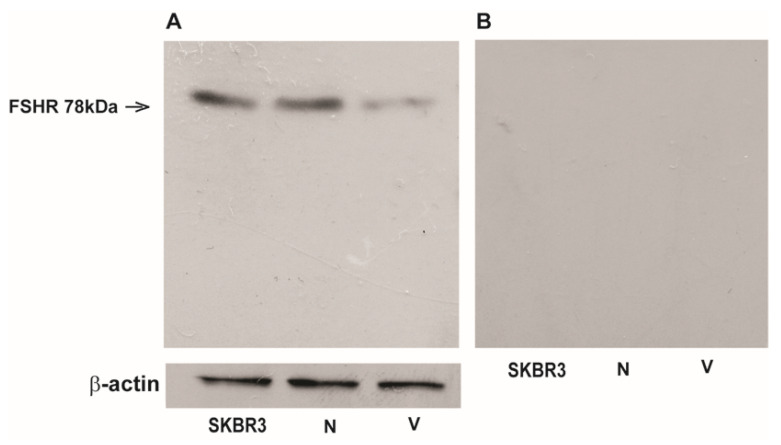
FSH-R expression is lower in human sperm from varicocele patients. (**A**) Immunoblot of FSH-R from a representative experiment of a human sperm sample (lane N) from a normozoospermic patient, and human sperm sample (lane V) from a varicocele patient; SK-BR-3 breast cancer cell line extract was used as positive control. (**B**) Immunoblot with the same loaded samples but without primary Abs and used to verify the Abs sensitivity as reported in Materials and Methods. β-actin served as a loading control. The number given on the left represents the FSH-R molecular weight.

To date, the presence of FSH-R in sperm is controversial. Herein, we first investigated the expression of FSH-R in ejaculated sperm from normozoospermic (N) and varicocele (V) patients by Western blot analysis. Both the anti-FSH-R Ab (6E8.2F5) and the anti-FSH-R (H-190) Abs were used and the same results were obtained. One band at 78 kDa was detected in normozoospermic samples, corresponding to the isoform long or the full-length G protein-coupled FSH-R (FSH-R-1). Interestingly, in sperm from varicocele patients the band was strongly reduced with respect to normal samples (Figure 6A). The band was not detected by NMR or NRM (Figure 6B) indicating that these antibodies were specific for FSH-R.

### 3.7. Immunofluorescence Assay

**Figure 7:**
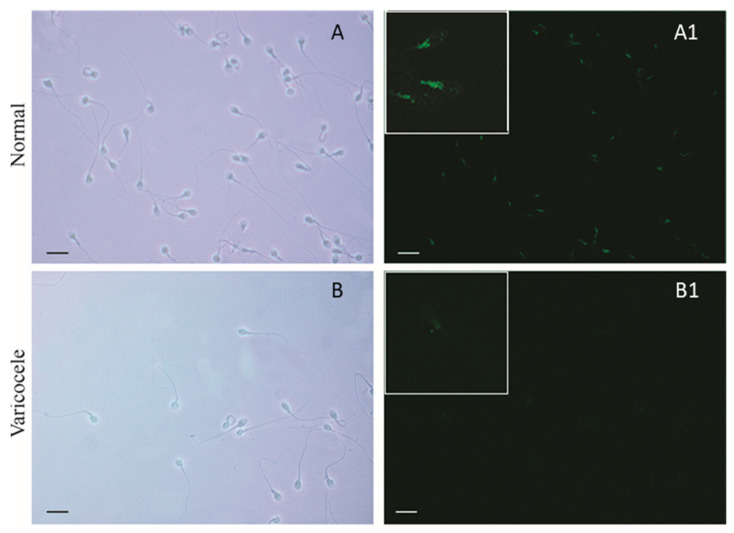
Immunofluorescence of FSH-R in human spermatozoa from normozoospermic (**A**,**A1**) and varicocele patients (**B**,**B1**). On the right, a bright green light showed FSH-R localization prevalently in the midpiece, which was significantly reduced in varicocele sperm. The inserts represent the higher magnification of the same samples. On the left the same images were shown in a bright field. Scale bars = 12.5 μm.

The immunofluorescence assay performed with the same antibodies used for WB evidenced a strong FSH-R localization in the midpiece and in the subequatorial regions (Figure 7A1). The immunoreaction was significantly reduced in varicocele sperm (Figure 7B1) and absent in the negative controls (see the inserts in Figure 7A1,B1).

### 3.8. FSH-Induced Activation of Akt is Mediated by PKA in Human Sperm

**Figure 8:**
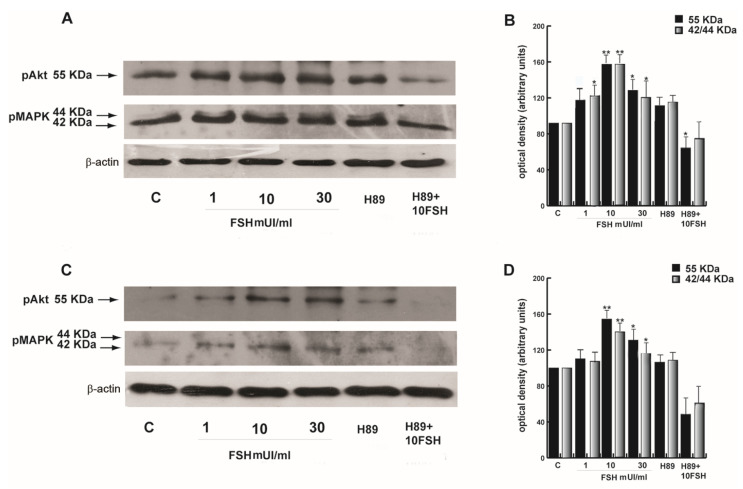
FSH induced the activation of pAkt and pMAPK by PKA in human sperm. (**A**) Representative immunoblot performed in normozoospermic human sperm treated as indicated in the figure. (**C**) Representative immunoblot performed in varicocele sperm treated as indicated in the figure. (**B**,**D**) The columns represent the band intensities, evaluated in terms of arbitrary densitometric units and presented as the mean ± SEM. * *P* < 0.05; ** *P* < 0.005 versus controls.

FSH-R is a glycosylated transmembrane protein that belongs to the G protein-coupled receptor family. Upon FSH binding, the activity of the FSH-R receptor is mediated by G proteins that activate the PKA pathway [39], which in turn induces the activation of the PI3K-AKT and MAPK42/44 signaling pathways [40]. Therefore, we studied the mechanisms involved in our cell type by testing increasing FSH concentrations. In normozoospermic samples both pAkt and pMAPK42/44 were induced particularly at 10 and 30 mUI/mL (Figure 8 A,B). In the same experimental conditions, the varicocele samples showed an activation of Akt and MAPK42/44 at 10 and 30 mUI/mL, although to a lesser extent with respect to the normozoospermic sperm (Figure 8 C,D). To analyze whether FSH effects occurred through the PKA kinase, we used 5 μM H89, a selective antagonist of this pathway, alone or combined with 10 mUI/mL FSH. H89 alone had no effect, whereas the combined treatment with 10 mUI/mL FSH showed lower values with respect to the controls, although not in a significant manner.

### 3.9. FSH-R Inhibition Abrogates the Stimulatory Effect of FSH on Human Sperm Motility and Survival

**Figure 9:**
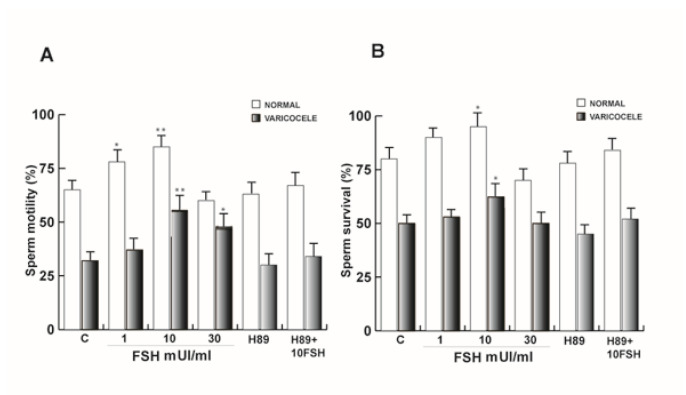
FSH-R influenced sperm motility and survival in normal and varicocele samples. (**A**) Sperm motility and (**B**) sperm vitality upon 1, 10, and 30 mUL/mL FSH, and H89 alone or combined with 10 mUI/mL FSH. Columns represent mean ± SEM of six independent experiments each done in duplicate. * *P* < 0.05 versus control, ** *P* < 0.001 versus controls.

Sperm motility is a distinctive parameter to measure semen quality, describing the ability of sperm to move properly towards an egg. Therefore, we next investigated the effect of increasing concentration of FSH as well as its combination with H89 on this important sperm feature. As shown in Figure 9A, sperm motility was significantly enhanced at 1 and 10 mUI/mL FSH treatment, whereas the co-treatment with 5 μM H89 reduced the effect. Varicocele sperm were responsive prevalently to the 10 and 30 mUI/mL FSH treatments, and these effects were lower with respect to normozoospermic sperm.

Another important hallmark of human sperm performance consists of its capacity to survive as much as possible to have the chance to find and fertilize the oocyte. In the same experimental conditions mentioned above, 10 mUI/mL FSH treatment had similar action both in normozoospermic and varicocele samples on sperm survival (Figure 9B).

### 3.10. FSH Induced Capacitation in Human Sperm

**Figure 10:**
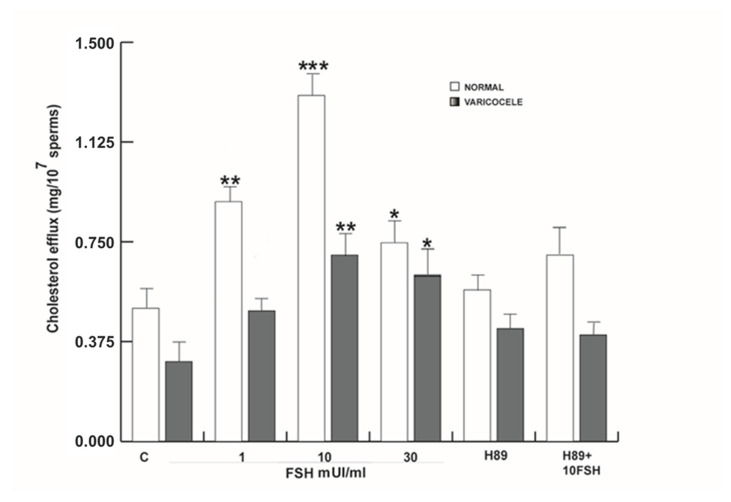
FSH induced capacitation in human sperm. Sperm cholesterol efflux in normal and varicocele samples upon 1, 10, and 30 mUL/mL FSH, and H89 alone or combined with 10 mUI/mL FSH. Columns represent mean ± SEM of six independent experiments each done in duplicate. * *P* < 0.05; ** *P* < 0.005; *** *P* < 0.001 versus controls.

In vivo, functional human sperm maturation, during which it acquires the competence to fertilize, occurs in the female reproductive tract, where spermatozoa undergo several modifications, collectively indicated as capacitation and including the membrane cholesterol efflux [41]. In somatic cells, FSH has an important role in cholesterol homeostasis. Therefore, this action may be important in sperm. Sperm from normal and varicocele samples were treated as indicated in Materials and Methods and centrifuged. The upper phase of the sample was used to determinate the cholesterol levels. As showed in Figure 10, the 1, 10, and 30 mUI/mL FSH treatments enhanced the cholesterol efflux significantly in normozoospermic samples depending on the concentration used. In varicocele samples, 10 and 30 mUI/mL FSH treatments were able to induce this action. In both sperm samples, the combined 10 mUI/mL FSH plus H89 treatment reversed, at least in part, the effect, suggesting an involvement of FSH in the induction of sperm capacitation.

### 3.11. FSH Increased Acrosome Reaction in Human Sperm

**Figure 11:**
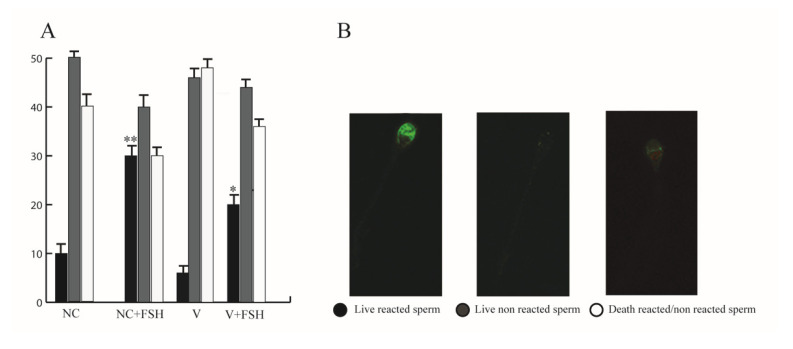
FSH increased the acrosome reaction in human sperm. Acrosome reaction (**A**) in normozoospermic and in varicocele samples with or without 10 mUI/mL FSH and (**B**) the staining pattern with FITC–PNA + PI. Columns represent mean percentage ± SEM of six independent experiments. * *P* < 0.05, ** *P* < 0.001 versus controls.

The membrane cholesterol efflux is an important step in initiating transmembrane signaling events to complete the sperm functional maturation process [41], which renders more fluid to the membrane, preparing sperm for the acrosome reaction. The latter process allows the sperm to fertilize. In the acrosome reaction assay we chose to treat only with the 10 mUI/mL FSH since this concentration induced the sperm activities that we have investigated in both normal and varicocele sperm. As showed in Figure 11A, the acrosome reaction significantly increased with respect to untreated sperm and, as expected, in a lower manner in varicocele sperm.

## 4. Discussion

In humans, FSH-Rs in normal conditions were found to be expressed in zona granulosa cells of the ovary and in Sertoli cells of the testis, where FSH promotes their growth and maturation [42,43]. Very recently, FSH-R expression was detected in a wide range of tumor samples [15]. Herein, we investigated for the first time FSH-R expression in the testis, ductuli efferentes, and proximal caput epididymis of the normal human male genital system and in human neoplastic testicular tissues. Interestingly, we found that the receptor is also present in human ejaculated spermatozoa and a different expression was noted in samples from patients with varicocele with respect to those of healthy men.

Recently, the expression of FSH-R has been observed in extragonadal tissues such as uterus, prostate, bone, and ovarian surface epithelial cells [4,23,44,45]. A low level of expression of FSH-R has also recently been identified in human osteoclasts and monocytes [46]. From these observations, it emerges that the expression of FSH-R is very cell specific, complicating the comprehension of the role of FSH as well as the cell response to FSH in these unsuspected targets.

In our study, first by using IHC analyses we investigated a detailed FSH-R localization in the different compartments of the human testis. In the Sertoli cells, it is located at the membrane and in some of these cells it appeared also at the cytoplasmic level. For many years, FSH-R localization was believed to be restricted to the plasma membrane. Subsequently, some findings detected FSH-R in Sertoli cell cytoplasm also by immunocytochemical procedures, and others were able to reveal the presence of endocytotic vesicles by immunocytochemistry [47]. FSH-R has been consistently found in the Sertoli cytoplasm, both in rats and in men [48,49], and in these cells, FSH-R internalization and degradation after endocytosis were demonstrated [50,51]. As it concerns the presence of FSH-R in germinal cells, it is controversial or, in humans, often excluded. Interestingly, germinal cells presenting the highest amount of positivity ranged from spermatogonia or spermatocytes up to round spermatids. The authors concluded that FSH is present in Sertoli cells and in round germinal cells (both expressing FSH-R), penetrating in a similar way in both kind of cells by endocytosis [26]. In the interstitial compartment of human testis, FSH-R was absent in the Leydig cells, in agreement with what was reported by the Human Protein Atlas [52].

Our findings also examined the presence of FSH-R in human ductuli efferentes and caput epididymis. Ductuli efferentes are known to reabsorb more than 90% of rete testis fluid and concentrate sperm as well as luminal components before entering the lumen of the epididymis [53,54]. We evidenced by IHC that the receptor is mainly in the ductuli efferentes with respect to the caput epididymis. In a previous study, FSH-R expression in epithelium of ductuli efferentes and along the human and rat epididymis was observed [55]. In humans, the distribution of FSH-R in the ductuli efferentes was observed in the cytoplasm of non-ciliated and ciliated cells. The authors also obtained an immunoreaction for FSH-R that was more intensive in the apical cytoplasm of all the principal cells of the caput epididymis [55]. Although these results are not fully in agreement with ours, we have to consider involvement of FSH-R in the functions of these male genital ducts. For instances, in FSH-R knock-out (FSHRKO) mice, morphological changes of the epididymis were noted. It appeared to be normal, but the tubular diameter of the cross-sections was smaller with respect to that observed in the wild type [56,57,58].

Recently, it has been shown that FSH-R is expressed by the endothelium of intra- and peri-tumoral blood vessels in a wide range of tumors such as lung, breast, prostate, colon, kidney, and urothelial carcinoma [14]. In contrast, the endothelia of vessels present in non-malignant tissues do not express FSH-R. It might be supposed that FSH-R-positive blood vessels represent the vasculature formed through tumoral neo-angiogenesis and therefore FSH-R may contribute to this process [59]. From a theoretical point of view, these findings are in agreement with the role of FSH-R in the regulation of angiogenesis, as reported for the ovary [60]. However, its functional role in tumors is currently under investigation.

In our study, we examined two types of testicular cancers: SE and the non-seminomatous germ cell tumor, EC. They present distinctive clinical features, differing for therapy and prognosis since EC tends to be metastatic at presentation and has a worse prognosis than SE. Overexpression of FSH-R was observed by IHC in EC samples and these data were confirmed by Western blot analyses, with respect to SE. Altogether, these observations may indicate that the high expression of the receptor in some cancers is involved in their progression. To corroborate this hypothesis, we compared by IHC the expression of ERβ, in both SE and EC, which we previously showed in SE [17,38], observing that the receptor is absent in EC. On the contrary, FSH-R is greatly present in EC. Therefore, ERβ loss and higher FSH-R expression are associated with advanced testicular tumor stage.

Few studies have raised the possibility that germ cells may exhibit FSH-R. Furthermore, the presence of FSH-R in germinal cells such as in ejaculated spermatozoa is controversial. To address this gap, we focused on the expression as well as on the eventual actions of FSH-R in human ejaculated sperm from normozoospermic and varicocele patients.

Via Western blots for FSH-R we observed a band of 78 kDa, which was consistent with the reported full length or long isoform that was chosen as the “canonical” sequence of the receptor also known as FSH-R-1. A previous study using two different models (mice and human Sertoli cells) showed that FSH-R was expressed in Sertoli cells as well as in germinal cells [26]. The germ cells presenting the highest amount of positivity ranged from spermatogonia or spermatocytes up to round spermatids [26]. Interestingly, we showed that FSH-R expression was expressed in normal ejaculated spermatozoa, whereas it was significantly reduced in sperm from varicocele patients. These data were corroborated by immunofluorescence assays. Unfortunately, testicular varicocele represents one of the most common causes of male infertility and although this pathology has been extensively studied, the mechanisms by which it can influence male fertility are not fully defined yet. Our previous studies showed for the first time that this pathology induces damage in the human male gamete at the molecular level. In fact, we observed that sperm that appeared to have normal morphology may possess an alteration in the molecular pool, implying a different responsiveness in the oocyte microenvironment. For instance, a reduction of steroid receptors, such as estrogen and progesterone receptors in samples from patients with varicocele, caused impaired sperm activity [25].

As it concerns FSH-R localization, in this research it was observed mainly in the midpiece and in the principal piece of the tail. Studies on FSH-R have shed some light on the mechanisms that presumably lead to FSH-R activation and to the signal transductions involved. These mechanisms have been reported to regulate a number of intertwined signaling pathways, including engagement of distinct kinases such as PKA, protein kinase B/Akt, and MAPK 42/44. [61]. However, generally the main target that mediates FSH-dependent signaling is PKA. Interestingly, it has been shown that Akt and MAPK 42/44 pathways may by PKA kinase-independent as well as PKA kinase-dependent. [62]. It is important to point out that during capacitation all the above-mentioned pathways are activated. Therefore, we investigated FSH-induced signaling in uncapacitated sperm by using a specific PKA inhibitor, the H89, and our data showed that both pAkt and pMAPK 42/44 are activated in a PKA-dependent fashion. These pathways were less triggered upon FSH treatments in varicocele sperm. Worth noting is that FSH treatments had a different pattern of response on motility and cholesterol efflux related to the healthy status of spermatozoa. In fact, in normal sperm, 1 and 10 mUI/mL FSH were enough to induce the effect, which was reduced at 30 mUI/mL, whereas in varicocele sperm, 10 and 30 mUI/mL were needed to obtain sperm activation. This difference in response may be due to the reduced expression of FSH-R in the varicocele sperm. Sperm survival upon FSH had a significant increase at 10 mUI/mL in both normal and varicocele sperm. Our data on the acrosome reaction assay confirmed that FSH-R is implied in the acquisition of sperm fertilizing ability. Recently, our studies showed that the disease causes damage in sperm at the molecular level, opening a new chapter in the already multifaceted physiopathology of varicocele. Overall, it appears that FSH treatment acts both on spermatogenesis and on sperm directly. Therefore, the positive effects of FSH could be of great help for men with idiopathic infertility and couples who have difficulty conceiving a child naturally or during medical assisted reproduction. Treatment with FSH could improve sperm’s potential fertilization in normal and in varicocele sperm, thereby resolving some cases of idiopathic infertility.

In summary, we demonstrated the presence of FSH-R in the first tracts of the human male genital system, supporting action on the anatomical shape of sperm maturation. FSH-R expression may distinguish normal testicular tissues from TGCTs as well as indicate the more aggressive cancer type. The receptor is expressed in human ejaculated sperm, indicating a direct action on the functional sperm maturation by capacitation and acrosome reaction in the female genital system. The reduced expression of FSH-R in varicocele sperm may represent another important molecular alteration in human sperm, contributing to the detrimental effect of this pathology in the acquisition of sperm fertilization capacity.

## 5. Conclusions

Different FSH-R expression in testicular tumor tissues could be informative of histological TGCT type and therefore in clinical decision-making as well as in patient counseling. From our study, the importance of evaluating the male gamete molecular anatomy also emerged, given the reduced content of the receptor in varicocele patients and the altered sperm responsiveness in the human female reproductive tract. The translation of the latter data in the clinical surgery of testicular varicocele needs to be taken into account since to date controversies exist regarding the opportunity to intervene surgically. Collectively, FSH-R expression could be considered a molecular marker of testicular disorders, even if future studies are needed to confirm this hypothesis.

## Figures and Tables

**Table 1:** FSH-R in human genital ducts.

Normal Testes	Ductuli Efferentes	Proximal Epididymus
Germ cells +	Epithelial cells +++	Epithelial cells ++
Sertoli cells +++	Muscle cells +	Muscle cells +
Leydig cells −	Stromal cells −	Stromal cells −

Staining intensity scores has been considered as it follows: − negative; + weak; ++ moderate; +++ strong.

**Table 2:** Immunostaining scores (Allred score median) of FSH-R and ERb in human seminoma and embryonal carcinoma samples.

	FSH-R	ERb
Seminoma	1	8
Embryonal carcinoma	6 **	4 *

Immunostained slides scores as follows: Total score = Proposition score + Intensity score (range 0–8). * *p* < 0.005 (one-way ANOVA test) versus Seminoma; ** *p* < 0.001 (one-way ANOVA test) versus Seminoma.
